# Changes in the Frequency and Type of Barriers to Reproductive Health Care Between 2017 and 2021

**DOI:** 10.1001/jamanetworkopen.2023.7461

**Published:** 2023-04-10

**Authors:** Aliza Adler, M. Antonia Biggs, Shelly Kaller, Rosalyn Schroeder, Lauren Ralph

**Affiliations:** 1 Maternal, Adolescent and Child Health Division, School of Public Health, University of California, Berkeley; 2 Advancing New Standards in Reproductive Health, Bixby Center for Global Reproductive Health, Department of Obstetrics, Gynecology, and Reproductive Sciences, University of California, San Francisco

## Abstract

**Importance:**

Previous research has documented individual-level barriers to reproductive health services, but few studies have examined national trends.

**Objective:**

To determine whether the number and type of barriers to reproductive health care experienced by US women of reproductive age changed from 2017 to 2021.

**Design, Setting, and Participants:**

This cross-sectional study used serial survey data, weighted to be nationally representative, collected in August 2017 and December 2021 from members of Ipsos’s KnowledgePanel who were aged 18 to 49 years and assigned female at birth.

**Exposures:**

Having experienced barriers to reproductive health care over the past 3 years.

**Main Outcomes and Measures:**

The primary outcome was number and type of barriers to reproductive health care services, including Papanicolaou tests or birth control, experienced in the past 3 years. Increases in barriers to reproductive health care were measured using multivariable linear regressions adjusted for age, race and ethnicity, education level, employment status, metropolitan area, geographic region, household income, and language (English vs Spanish).

**Results:**

Of 29 496 KnowledgePanel members invited, 7022 (mean [SD] age, 33.9 [9.0] years) and 6841 (mean [SD] age, 34.2 [8.6] years) completed the 2017 and 2021 surveys (50% and 45% response rates, respectively). Among 12 351 participants who indicated that they had ever tried accessing reproductive health services, 35.9% (95% CI, 34.8%-37.0%) were aged 30 to 39 years; 5.5% (95% CI, 4.9%-6.2%) were Asian or Pacific Islander, 13.7% (95% CI, 12.8%-14.6%) were Black, 19.1% (95% CI, 18.1%-20.1%) were Hispanic, 58.2% (95% CI, 57.0%-59.5%) were White, and 3.5% (95% CI, 3.1%-4.0%) were multiracial or of other race or ethnicity; and 11.7% (95% CI, 11.0%-12.5%) were living below 100% of the federal poverty level. Covariate distribution was similar across years. In bivariable analyses, participants were more likely to report experiencing a given barrier in the past 3 years in 2021 than in 2017 for all but 2 barriers. More people experienced 3 or more barriers in 2021 (18.6%; 95% CI, 17.3%-20.0%) than in 2017 (16.1%; 95% CI, 14.9%-17.4%) (*P* = .008). In multivariable analyses, the mean number of barriers increased significantly from 1.09 (95% CI, 1.02-1.14) in 2017 to 1.29 (95% CI, 1.22-1.37) (*P* < .001) in 2021. Participants who were aged 25 to 29 years (0.42; 95% CI, 0.37-0.47), identified as Hispanic (0.41; 95% CI, 0.38-0.45), had no high school diploma or General Educational Development test (0.62; 95% CI, 0.53-0.72), lived below 100% of the federal poverty level (0.65; 95% CI, 0.55-0.73), and took the survey in Spanish (0.87; 95% CI, 0.73-1.01) saw the greatest increases in mean number of barriers between 2017 and 2021.

**Conclusions and Relevance:**

The study findings suggest that barriers to reproductive health care increased between 2017 and 2021, with the largest increases observed among individuals from historically disadvantaged populations. Efforts are needed to ensure that reproductive health care access remains a priority.

## Key Points

**Question:**

How have barriers to reproductive health care services changed between 2017 and 2021?

**Findings:**

In this cross-sectional study fielded in fall 2017 and winter 2021, an increase was observed in the proportion of US women of reproductive age reporting a barrier to accessing wanted reproductive health care services, as well as an increase in the number of barriers experienced. Participants from historically disadvantaged populations saw the greatest increase in number of barriers.

**Meaning:**

The study’s findings suggest that efforts are needed to ensure reproductive health care access, especially during disruptive events.

## Teaser

This cross-sectional study uses survey data to examine whether barriers to reproductive health care experienced by US women changed from 2017 to 2021.

## Introduction

Reproductive health care services are among the most common health care needs of women of reproductive age,^1^ and access to such care is of paramount public health importance. Delaying or forgoing reproductive health care not only can result in morbidity but also, in situations such as untreated sexually transmitted infections, can result in an increased risk of serious complications, such as infertility and pelvic inflammatory disease.^2^ More broadly, access to wanted reproductive health care, including contraceptive services and abortion, allows individuals to exercise their bodily autonomy and control if and/or when they want to have children, which can ultimately improve individuals’ well-being and quality of life.^2^ Previous studies have documented a wide variety of individual-level factors that pose barriers to accessing reproductive health services, which in turn can be detrimental to health outcomes.

Such documented barriers are wide ranging and include cost or lack of insurance, difficulty obtaining an appointment or reaching a clinic, not having a regular physician, and fear of lack of confidentiality of services.^3,4,5^ Difficulty accessing a clinic has been well documented as a leading, and potentially insurmountable, barrier to abortion^6^ and other reproductive health services, with previous research suggesting that challenges in making an appointment and/or reaching a clinic are primary barriers for many individuals seeking contraceptive services.^5^ Additionally, previous studies have highlighted that barriers to reproductive health services often disproportionately affect historically marginalized groups.^4,7,8,9^ Such individuals often experience many of the aforementioned barriers to a far greater extent.^7^ One study of adolescents found that individuals who identified as a gender and/or sexual minority or as Asian or Pacific Islander/Native Hawaiian were more likely to report experiencing 5 or more barriers to reproductive health services than the reference group.^4^ The same study found that lesbian, gay, bisexual, transgender, queer (or questioning), and other (LGBTQ+) adolescents were more likely to report cost and confidentiality as barriers to care than non-LGBTQ+ youths.^4^ As barriers to reproductive health services can result in delays in or inability to receive care, it is important to assess and track national trends in the numbers and types of barriers being experienced by individuals of reproductive age.

While previous studies have documented the individual factors associated with experiencing barriers to reproductive health services, few have focused on the broader policy context or national trends surrounding such barriers.^3,4,5,10,11^ Furthermore, many studies primarily focused on barriers experienced by adolescents^3,4,9^ rather than all individuals of reproductive age. Still fewer focused on a broad spectrum of reproductive health services, such as Papanicolaou tests or sexually transmitted infection testing and treatment,^9^ with most focusing on contraceptive access.^12^ Of the studies that focused on policy changes, some evaluated outcomes associated with delivery of services from the perspective of clinics and clinicians rather than the experiences of patients accessing care.^13^ No studies have been published to our knowledge that were nationally representative or compared similarly representative study populations over 2 periods.

The objective of our study was to describe changes in barriers and access to a broad spectrum of reproductive health services from individuals’ perspectives using serial, nationally representative, cross-sectional surveys fielded in August 2017 and December 2021. We hypothesized that due to factors such as COVID-19 and increasing federal restrictions on reproduction health services,^14,15^ barriers to reproductive health services would increase over this period, with historically marginalized groups, including minoritized racial and ethnic groups and individuals with lower incomes, experiencing the greatest impact.

## Methods

### Sample

This serial cross-sectional study used surveys administered in 2017 and 2021 by Ipsos (formerly the GfK Group) to members of its online KnowledgePanel.^16^ Ipsos uses probability-based sampling techniques of all US addresses to recruit panel members so that the sample can be weighted to be representative of the noninstitutionalized US population based on US census data. Panel members are regularly invited to complete online surveys and are provided with the technology to do so, if needed.

In August 2017 and December 2021, Ipsos invited eligible panel members to complete a cross-sectional survey on reproductive health care experiences and opinions designed by researchers at the University of California, San Francisco. Eligibility was restricted to panel members who indicated that they were assigned female at birth and were aged 18 to 49 years. Survey administration was comparable across survey years. Invited participants received reminders to complete the survey 3 and 8 days after the initial invitation, and data collection closed when sample size targets (approximately 7000 responses) were met. Data collection took 15 days in 2017 and 32 days in 2021. Study participants received compensation through Ipsos’s points program, ranging from $4 to $6 per month, depending on participation. This study was approved by the University of California, San Francisco, institutional review board, and study participants provided electronic informed consent before taking the survey. This study followed the Strengthening the Reporting of Observational Studies in Epidemiology (STROBE) guidelines.^17^

### Measures

Our primary outcome of interest was barriers to reproductive health services experienced in 2014-2017 and 2018-2021. Reproductive health services were described to participants as “a Pap smear, which is a test to check for cervical cancer, or family planning, like birth control methods.” Participants who had ever tried accessing reproductive health services were asked a follow-up question about specific types of barriers from a predefined list where they could select all that applied. Barriers were identical, with the exception of 1 additional type in the 2021 survey: “get services without having to tell people you didn’t want to tell.” For analyses and variable creation (detailed next), only the 9 barriers asked consistently across both surveys were used.

We created a continuous variable for the number of barriers experienced for our primary outcome. Responses could range from 0 (no barriers) to 9 (all barriers). We also generated a 4-part categorical variable that included 0, 1, 2, or 3 or more barriers. Two investigators (A.A. and L.R.) independently reviewed the list of 9 barriers and collapsed them into 5 domains: cost, access, logistical challenges, interpersonal relationships, and privacy.

Ipsos routinely collects sociodemographic information for KnowledgePanel members (eTable in Supplement 1). In this analysis, we used the following Ipsos-collected variables: age (years), self-selected race and ethnicity, highest level of education completed, employment status, metropolitan statistical area, household income, and sexuality (only collected in 2021). We based geographic region on state of residence and generated a 4-part categorical variable denoting the participant’s state of residence expansion, or lack thereof, of Medicaid eligibility under the Patient Protection and Affordable Care Act (ACA), as well as whether the state opted into expansion of Medicaid coverage for family planning services for individuals not eligible for traditional Medicaid.^18^ By using household income and size, we calculated household percentage of the federal poverty level (FPL) using 2017 census thresholds for 2017 data and 2021 thresholds for 2021 data.^19,20^

### Statistical Analysis

All analyses used survey weights generated by Ipsos and were designed to weight the sample to represent benchmarks on race and ethnicity, age, education, census region (Northeast, Midwest, South, West), and metropolitan status (yes, no) for the most recently available US Census Bureau Current Population Survey. We used descriptive statistics for participant characteristics in 2017 and 2021, followed by χ^2^ tests to evaluate whether there were any differences in the distribution of covariates across survey years. We then generated weighted frequencies to summarize the proportion of the sample who experienced each specific barrier by survey year, as well as the mean number of barriers, categorical number of barriers, and each barrier domain. To evaluate whether the mean number of barriers experienced had changed between 2017 and 2021, we calculated the difference in means between 2017 and 2021 and tested whether values significantly differed over time using weighted linear regression models with number of barriers to reproductive health services as the dependent variable and year of the survey as an independent variable. Linear regression is reasonable due to large sample sizes, and while estimated values may be less than 0 or larger than 9, the large sample size lessens concerns of this limitation. We also calculated the difference in the weighted percentage for each individual barrier between years and ran logistic regressions to determine whether the difference was significant. Change over time was considered significant if the *P* value on year of the survey was 0.05 or less in these models.

We then conducted a series of multivariable linear regressions to determine whether the mean number of barriers to reproductive health services had increased within specific groups, while adjusting for covariates. Covariates included age, race and ethnicity, highest level of education, percentage of the FPL, metropolitan statistical area, geographic region, living in a state that implemented ACA Medicaid expansion, and language in which the survey was taken. All covariates were selected a priori based on characteristics known to be associated with barriers to health care services from the literature.^4,14^ All analyses were conducted using Stata, version 14 (StataCorp LLC) statistical software.

## Results

Of 14 151 panel members approached for the 2017 survey, 7022 (mean [SD] age, 33.9 [9.0] years) completed the survey (50% response rate). In 2021, of 15 345 adult panel members approached, 6841 (mean [SD] age, 34.2 [8.6] years) completed the survey (45% response rate). We restricted the combined sample to participants who indicated that they had ever tried accessing reproductive health services and were thus asked questions regarding barriers to reproductive health care, leaving a combined sample size of 12 351 (89% of combined sample) participants (Figure).

**Figure.**
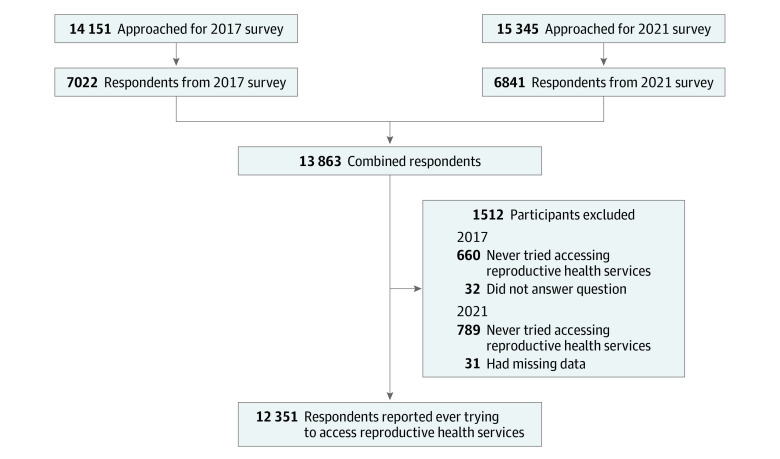
Sample Selection Flow Diagram

The data are weighted to be nationally representative, and the distribution of covariates was largely similar between survey years (Table 1). In our combined data set with responses from 2017 and 2021, 35.9% (95% CI, 34.8%-37.0%) of respondents were aged 30 to 39 years; 5.5% (95% CI, 4.9-6.2%) were Asian or Pacific Islander, 13.7% (95% CI, 12.8%-14.6%) were Black, 19.1% (95% CI, 18.1%-20.1%) were Hispanic, 58.2% (95% CI, 57.0%-59.5%) were White, and 3.5% (95% CI, 3.1%-4.0%) were multiracial or of other race or ethnicity; 31.0% (95% CI, 29.9%-32.1%) had completed some college or received an associate’s degree; 92.3% (95% CI, 91.6%-92.9%) completed the survey in English; 11.7% (95% CI, 11.0%-12.5%) were living below 100% of the FPL; and 88.1% (95% CI, 87.3%-88.8%) were living in metropolitan areas. Most participants lived in the South Atlantic region (20.4%; 95% CI, 19.4%-21.4%) and in states that implemented the ACA Medicaid expansion with family planning services (43.8%; 95% CI, 42.6%-45.0%) (Table 1).

**Table 1.** Demographic Characteristics of 12 351 Participants Who Ever Tried to Access Reproductive Health Services by Survey Year

Characteristic	2017		2021		Weighted *P* value^a^
	Unweighted No. (n = 6330)	Weighted % (95% CI)	Unweighted No. (n = 6021)	Weighted % (95% CI)	
Age, y					
18-24	329	12.7 (11.3-14.2)	264	11.8 (10.4-13.3)	.66
25-29	960	17.1 (15.9-18.4)	876	17.2 (15.9-18.6)	
30-39	2519	35.6 (34.1-37.1)	2431	36.9 (35.2-38.5)	
40-49	2522	34.7 (33.2-36.2)	2450	34.1 (32.5-35.8)	
Race and ethnicity					
Asian or Pacific Islander	194	6.1 (5.2-7.2)	184	5.0 (4.2-5.9)	<.001
Black	608	13.4 (12.2-14.8)	499	14.0 (12.7-15.4)	
Hispanic	1199	19.5 (18.2-20.9)	1079	18.5 (17.1-20.0)	
White	4099	58.4 (56.7-60.1)	4051	58.0 (56.2-59.8)	
Multiracial or other^b^	230	2.6 (2.1-3.1)	208	4.5 (3.8-5.3)	
Highest level of education					
No high school diploma or GED	238	9.4 (8.2-10.8)	204	7.3 (6.3-8.5)	<.001
High school graduate (high school diploma)	853	20.2 (18.8-21.7)	675	18.4 (17.0-20.0)	
Some college or associate’s degree	2073	32.1 (30.7-33.7)	1653	29.7 (28.1-31.4)	
Bachelor’s degree	2029	23.4 (22.2-24.7)	2119	25.5 (24.1-27.0)	
Master’s degree or above	1137	14.9 (13.8-15.9)	1370	19.0 (17.8-20.3)	
Percentage of the FPL					
<100	1045	13.7 (12.7-14.8)	837	9.4 (8.5-10.5)	<.001
100-199	1110	16.1 (14.9-17.4)	927	12.8 (11.7-14.0)	
≥200	4175	70.2 (68.7-71.6)	4257	77.7 (76.3-79.1)	
Metropolitan statistical area					
Nonmetropolitan	753	12.6 (11.5-13.7)	790	11.1 (10.1-12.2)	.07
Metropolitan	5577	87.4 (86.3-88.5)	5231	88.8 (87.8-89.9)	
Geographic region					
New England^c^	247	4.1 (3.5-4.8)	247	4.5 (3.8-5.3)	.61
Middle Atlantic^d^	776	12.4 (11.4-13.6)	664	12.2 (11.0-13.4)	
East North Central^e^	1048	13.6 (12.6-14.7)	1036	14.1 (13.0-15.3)	
West North Central^f^	536	6.6 (5.9-7.4)	547	6.5 (5.8-7.4)	
South Atlantic^g^	1183	20.4 (19.1-21.8)	1123	20.2 (18.8-21.7)	
East South Central^h^	306	5.3 (4.6-6.2)	336	5.9 (5.1-6.8)	
West South Central^i^	711	12.6 (11.5-13.8)	675	11.8 (10.8-13.0)	
Mountain^j^	487	7.3 (6.5-8.2)	511	8.2 (7.3-9.3)	
Pacific^k^	1036	17.7 (16.4-19.0)	882	16.4 (15.1-17.8)	
State implementation of ACA Medicaid expansion pathway and family planning–only programs					
Expansion with family planning (19 states^l^)	2660	44.1 (42.4-45.7)	2482	43.7 (42.0-45.5)	.79
Expansion with no family planning (18 states^m^ and the District of Columbia)	1647	23.1 (21.8-24.5)	1627	24.1 (22.7-25.6)	
No expansion with family planning only (9 states^n^)	1684	27.9 (26.5-29.4)	1593	27.3 (25.7-28.9)	
No expansion and no family planning (4 states^o^)	339	4.9 (4.2-5.7)	319	4.9 (4.2-5.7)	
Language survey taken in					
English	5810	90.9 (89.8-91.8)	5689	93.9 (92.9-94.7)	<.001
Spanish	520	9.1 (8.2-10.2)	332	6.1 (5.3-7.1)	
Sexuality^p^					
Gay or lesbian	NA	NA	95	1.8 (1.4-2.4)	NA
Heterosexual	NA	NA	5219	86.7 (85.4-87.9)	
Bisexual	NA	NA	441	7.0 (6.1-8.0)	
Queer	NA	NA	84	1.3 (0.9-1.8)	
Other	NA	NA	103	1.8 (1.3-2.3)	
No response or missing data	NA	NA	79	1.5 (1.1-2.0)	
Employment status					
Working full time	4729	73.1 (71.5-74.5)	3573	58.5 (56.7-60.2)	<.001
Working part time	414	7.4 (6.5-8.5)	1071	16.8 (15.5-18.2)	
Not working	1187	19.5 (18.2-20.9)	1377	24.7 (23.1-26.3)	

Abbreviations: ACA, Patient Protection and Affordable Care Act; FPL, federal poverty level; GED, General Educational Development test; NA, not applicable.

^a^ χ^2^ Test of weighted percentages across years.

^b^ Other race and ethnicity included American Indian or Alaska Native, Chinese, Filipino, Japanese or Korean, Southeast Asian, 2 or more races or ethnicities listed as other.

^c^ Connecticut, Maine, Massachusetts, New Hampshire, Rhode Island, Vermont.

^d^ New Jersey, New York, Pennsylvania.

^e^ Indiana, Illinois, Michigan, Ohio, Wisconsin.

^f^ Iowa, Kansas, Minnesota, Missouri, Nebraska, North Dakota, South Dakota.

^g^ Delaware, District of Columbia, Florida, Georgia, Maryland, North Carolina, South Carolina, Virginia, West Virginia.

^h^ Alabama, Kentucky, Mississippi, Tennessee.

^i^ Arkansas, Louisiana, Oklahoma, Texas.

^j^ Arizona, Colorado, Idaho, Montana, Nevada, New Mexico, Utah, Wyoming.

^k^ Alaska, California, Hawaii, Oregon, Washington.

^l^ California, Connecticut, Indiana, Louisiana, Maine, Maryland, Minnesota, Montana, New Hampshire, New Mexico, New Jersey, New York, Oklahoma, Oregon, Pennsylvania, Rhode Island, Vermont, Virginia, Washington.

^m^ Alaska, Arizona, Arkansas, Colorado, Delaware, Hawaii, Idaho, Illinois, Iowa, Kentucky, Massachusetts, Michigan, Nebraska, Nevada, North Dakota, Ohio, Utah, West Virginia.

^n^ Alabama, Florida, Georgia, Mississippi, North Carolina, South Carolina, Texas, Wisconsin, Wyoming.

^o^ Kansas, Missouri, South Dakota, Tennessee.

^p^ Not asked in 2017 survey.

The proportion of participants who had ever accessed reproductive health services was similar in 2017 (84.9%; 95% CI, 83.4%-86.2%) and 2021 (84.1%; 95% CI, 82.7%-85.4%). In bivariable analysis, the weighted frequencies of participants experiencing a given barrier significantly increased between 2017 and 2021 for all but 2 barriers: difficulty paying for reproductive health services significantly decreased (difference, −1.9%; 95% CI, −2.0% to −1.85%; *P* = .046), and finding a physician or clinic that accepts one’s insurance had no significant change (difference, 0.9%; 95% CI, 0.8%-1.0%; *P* = .33) (Table 2). The largest increase in participants reporting experiencing a given barrier was for finding a physician or clinic where they felt comfortable (difference, 4.9%; 95% CI, 4.7%-5.2%; *P* < .001) (Table 2).

**Table 2.** Unadjusted Frequencies of Adult Participants Experiencing Barriers to RH Services by Survey Year, Weighted Analysis

	2017		2021		Differences in weighted % between years (95% CI)	RR (95% CI)	*P* value^a^
	Unweighted No.	Weighted % (95% CI)	Unweighted No.	Weighted % (95% CI)			
Ever tried accessing RH services (n = 13 844)	6330	84.9 (83.4 to 86.2)	6021	84.1 (82.7 to 85.4)	−0.8 (−0.7 to −0.8)	NA	.44
Experienced any barriers to RH services (n = 12 351)	2647	40.6 (39.0 to 42.2)	2774	44.6 (42.8 to 46.3)	4.0 (3.8 to 4.1)	NA	.001
Specific barriers accessing RH services in past 3 y							
(1) Get time off work or school to go to physician or clinic	1250	19.1 (17.8 to 20.4)	1436	24.2 (22.7 to 25.8)	5.1 (4.9 to 5.4)	NA	<.001
(2) Find a physician or clinic where you felt comfortable	1267	19.9 (18.6 to 21.23	1542	24.8 (23.3 to 26.4)	4.9 (4.7 to 5.2)	NA	<.001
(3) Find services with people who speak the same language as you	266	4.5 (3.8 to 5.3)	410	7.9 (6.9 to 8.9)	3.4 (3.1 to 3.6)	NA	<.001
(4) Find transportation to get to a physician or clinic	490	7.9 (7.1 to 8.9)	583	10.5 (9.4 to 11.7)	2.8 (2.6 to 3.1)	NA	.001
(5) Find child care so you could go to a physician or clinic	660	10.8 (9.8 to 11.9)	814	13.2 (12.1 to 14.4)	2.4 (2.3 to 2.5)	NA	.002
(6) Find a physician or clinic that offers RH services	508	8.7 (7.7 to 9.7)	606	10.5 (9.4 to 11.7)	1.8 (1.7 to 2.0)	NA	.02
(7) Your partner or someone in your family did not want you to go	192	3.0 (2.5 to 3.7)	234	4.5 (3.8 to 5.3)	1.5 (1.3 to 1.6)	NA	.003
(8) Find a physician or clinic that accepts your insurance	967	15.4 (14.2 to 16.7)	1003	16.3 (15.0 to 17.7)	0.9 (0.8 to 1.0)	NA	.33
(9) Pay for RH services	1308	19.2 (17.9 to 20.5)	1124	17.3 (16.0 to 18.6)	−1.9 (−2.0 to −1.85)	NA	.046
(10) Get services without having to tell people you did not want to tell	Not asked in 2017	Not asked in 2017	476	9.3 (8.2 to 10.4)	Not asked in 2017	NA	Not asked in 2017
No. of barriers experienced^b^							
0	3671	59.3 (57.6 to 60.9)	3238	55.2 (53.5 to 57.0)	−4.1 (−4.3 to −4.0)	[Reference]	
1	1027	15.3 (14.2 to 16.5)	999	15.7 (14.5 to 17.0)	0.4 (0.3 to 0.5)	1.02 (0.99 to 1.06)	
2	590	9.1 (8.2 to 10.2)	639	10.2 (9.2 to 11.3)	1.2 (1.0 to 1.1)	1.05 (1.00 to 1.09)	
≥3	1030	16.1 (14.9 to 17.4)	1136	18.6 (17.3 to 20.0)	2.5 (2.4 to 2.6)	1.06 (1.02 to 1.09)	
Did not answer	12	0.2 (0.1 to 0.5)	9	0.2 (0.1 to 0.5)	−0.016 (−0.02 to 0)	1.00 (0.75 to 1.34)	
Mean (95% CI)^b^	1.09 (1.05 to 1.14)	1.09 (1.02 to 1.15)	1.29 (1.24 to 1.34)	1.29 (1.22 to 1.37)	0.20 (0.20-0.22)^c^	NA	<.001^d^
Barrier domains							
Cost^e^	1684	25.3 (23.9 to 26.8)	1551	24.7 (23.2 to 26.3)	−0.6 (−0.7 to −0.5)	NA	.60
Access^f^	1403	22.1 (20.7 to 23.5)	1697	27.8 (26.2 to 29.4)	5.7 (5.4 to 5.9)	NA	<.001
Logistical challenges^g^	1668	25.6 (24.2 to 27.1)	1901	31.2 (29.6 to 32.9)	5.6 (5.4 to 5.8)	NA	<.001
Interpersonal relationships^h^	192	3.0 (2.5 to 3.7)	234	4.5 (3.8 to 5.3)	1.5 (1.3 to 1.6)	NA	.003
Privacy^i^	Not asked in 2017	Not asked in 2017	476	9.3 (8.2 to 10.4)	Not asked in 2017	NA	Not asked in 2017

Abbreviations: NA, not applicable; RH, reproductive health; RR, relative risk.

^a^ *P* value obtained from the year term in logistic regression.

^b^ RR obtained from multinomial regression; barrier “get services without having to tell people you did not want to” removed from analysis for consistency between years.

^c^ Difference in weighted mean.

^d^ Linear regression.

^e^ Combination of barriers (8) find a physician or clinic that accepts your insurance and (9) pay for RH services.

^f^ Combination of barriers (6) find a physician or clinic that offers RH services, (2) find a physician or clinic where you felt comfortable, and (3) find services with people who speak the same language as you.

^g^ Combination of barriers (4) find transportation, (1) get time off work, and (5) find child care.

^h^ Barrier (7) your partner or someone did not want you to go.

^i^ Barrier (10) get services without having to tell people you did not want to tell.

We found that more participants experienced 3 or more barriers in 2021 (18.6%; 95% CI, 17.3%-20.0%) than in 2017 (16.1%; 95% CI, 14.9%-17.4%) (*P* = .008) (Table 2). Significantly more participants experienced at least 1 barrier in 2021 (44.8%) than in 2017 (40.5%) (*P* = .001).

When examining weighted frequencies of domains of barriers, we also found significant increases between surveys for the following categories: access (difference, 5.7%; 95% CI, 5.4%-5.9%; *P* < .001), logistical challenges (difference, 5.6%; 95% CI, 5.4%-5.8%; *P* < .001), and interpersonal relationships (difference, 1.5%; 95% CI, 1.3%-1.6%; *P* = .003) between 2017 and 2021 (Table 2). Overall, the weighted mean number of barriers experienced by participants significantly increased from 1.09 (95% CI, 1.02-1.14) in 2017 to 1.29 (95% CI, 1.22-1.37) (*P* < .001) in 2021 (Table 3) when adjusting for covariates. Furthermore, we found the weighted mean number of barriers experienced to significantly increase across all but 9 of our 38 subgroup analyses (Table 3). By difference in weighted means between 2017 and 2021, participants who were aged 25 to 29 years (0.42; 95% CI, 0.37-0.4), identified as Hispanic (0.41; 95% CI, 0.38-0.45), had no high school diploma or General Educational Development test (0.62; 95% CI, 0.53-0.72), lived at less than 100% of the FPL (0.65; 95% CI, 0.55-0.73), and took the survey in Spanish (0.87; 95% CI, 0.73-1.01) saw the greatest increase in the mean number of barriers experienced (Table 2).

**Table 3.** Multivariable Linear Regression Models of the Number of Barriers Experienced Overall and Among Population Subgroups, Weighted Analysis

Characteristic	Weighted mean No. of barriers experienced (95% CI)		Difference in weighted means between years (95% CI)	*P* value^a^
	2017	2021		
Overall	1.09 (1.02 to 1.15)	1.29 (1.22 to 1.37)	0.20 (0.19 to 0.22)	<.001
Age, y				
18-24	1.36 (1.12 to 1.59)	1.50 (1.20 to 1.79)	0.14 (0.08 to 0.20)	.08
25-29	1.32 (1.15 to 1.49)	1.74 (1.53 to 1.96)	0.42 (0.37 to 0.47)	<.001
30-39	1.13 (1.03 to 1.23)	1.22 (1.11 to 1.33)	0.09 (0.08 to 1.00)	.005
40-49	0.83 (0.74 to 0.92)	1.08 (0.96 to 1.20)	0.25 (0.22 to 0.28)	.001
Race or ethnicity				
Asian or Pacific Islander	0.74 (0.51 to 0.97)	0.92 (0.62 to 1.23)	0.18 (0.11 to 0.26)	.29
Black	1.43 (1.21 to 1.65)	1.64 (1.38 to 1.91)	0.21 (0.17 to 0.26)	.04
Hispanic	1.55 (1.37 to 1.73)	1.96 (1.75 to 2.18)	0.41 (0.38 to 0.45)	<.001
White	0.88 (0.81 to 0.94)	1.03 (0.95 to 1.11)	0.15 (0.03 to 0.17)	<.001
Multiracial or other^b^	1.34 (0.99 to 1.68)	1.29 (0.98 to 1.60)	−0.05 (−0.01 to −0.08)	.29
Highest level of education				
No high school diploma or GED	2.07 (1.70 to 2.44)	2.69 (2.23 to 3.16)	0.62 (0.53 to 0.72)	.02
High school graduate (high school diploma)	1.29 (1.13 to 1.45)	1.65 (1.43 to 1.86)	0.35 (0.30 to 0.41)	.002
Some college or associate’s degree	1.16 (1.06 to 1.25)	1.24 (1.11 to 1.37)	0.08 (0.05 to 0.12)	.08
Bachelor’s degree	0.72 (0.65 to 0.79)	1.01 (0.89 to 1.12)	0.29 (0.24 to 0.33)	<.001
Master’s degree or above	0.62 (0.53 to 0.72)	0.88 (0.78 to 0.99)	0.26 (0.25 to 0.29)	<.001
Percentage of the FPL				
<100	2.12 (1.89 to 2.36)	2.77 (2.44 to 3.09)	0.65 (0.55 to 0.73)	.004
100-199	1.44 (1.29 to 1.59)	1.75 (1.53 to 1.98)	0.31 (0.24 to 0.39)	.02
≥200	0.80 (0.74 to 0.87)	1.04 (0.96 to 1.12)	0.24 (0.22 to 0.25)	<.001
Metropolitan statistical area				
Nonmetropolitan	1.12 (0.95 to 1.28)	1.30 (1.07 to 1.51)	0.18 (0.12 to 0.23)	.10
Metropolitan	1.08 (1.01 to 1.15)	1.29 (1.21 to 1.38)	0.21 (0.20 to 0.23)	<.001
Geographic region				
New England^c^	0.74 (0.54 to 0.94)	0.99 (0.72 to 1.26)	0.25 (0.18 to 0.32)	.03
Middle Atlantic^d^	0.83 (0.68 to 0.98)	1.31 (1.09 to 1.53)	0.48 (0.41 to 0.55)	<.001
East North Central^e^	1.06 (0.91 to 1.22)	1.11 (0.94 to 1.29)	0.05 (0.03 to 0.07)	.32
West North Central^f^	0.75 (0.59 to 0.92)	1.20 (0.88 to 1.51)	0.45 (0.29 to 0.59)	.02
South Atlantic^g^	1.19 (1.03 to 1.35)	1.36 (1.18 to 1.54)	0.17 (0.15 to 0.19)	.02
East South Central^h^	1.25 (0.92 to 1.58)	1.58 (1.17 to 1.99)	0.33 (0.25 to 0.41)	.04
West South Central^i^	1.35 (1.15 to 1.54)	1.39 (1.18 to 1.61)	0.04 (0.03 to 0.07)	.55
Mountain^j^	1.18 (0.95 to 1.42)	1.30 (1.03 to 1.56)	0.12 (0.08 to 0.14)	.09
Pacific^k^	1.09 (0.95 to 1.24)	1.30 (1.12 to 1.49)	0.21 (0.17 to 0.25)	.005
State implementation of ACA Medicaid expansion pathway and family planning–only programs				
Expansion with family planning (19 states^l^)	0.96 (0.87 to 1.04)	1.25 (1.14 to 1.36)	0.29 (0.27 to 0.32)	<.001
Expansion with no family planning (18 states^m^ and District of Columbia)	1.00 (0.88 to 1.12)	1.20 (1.05 to 1.34)	0.19 (0.18 to 0.22)	.002
No expansion with family planning only (9 states^n^)	1.37 (1.23 to 1.52)	1.39 (1.23 to 1.54)	0.02 (0 to 0.02)	.45
No expansion and no family planning (4 states^o^)	1.02 (0.70 to 1.34)	1.66 (1.21 to 2.11)	0.64 (0.51 to 0.77)	.01
Language survey taken in				
English	1.02 (0.95 to 1.08)	1.21 (1.13 to 1.28)	0.19 (0.18 to 0.20)	<.001
Spanish	1.77 (1.49 to 2.05)	2.64 (2.22 to 3.06)	0.87 (0.73 to 1.01)	<.001

Abbreviations: ACA, Patient Protection and Affordable Care Act; FPL, federal poverty level; GED, General Educational Development test.

^a^ All regression models controlled for the covariates listed in the characteristics column.

^b^ Other race and ethnicity included American Indian or Alaska Native, Chinese, Filipino, Japanese or Korean, Southeast Asian, 2 or more races or ethnicities listed as other.

^c^ Connecticut, Maine, Massachusetts, New Hampshire, Rhode Island, Vermont.

^d^ New Jersey, New York, Pennsylvania.

^e^ Indiana, Illinois, Michigan, Ohio, Wisconsin.

^f^ Iowa, Kansas, Minnesota, Missouri, Nebraska, North Dakota, South Dakota.

^g^ Delaware, District of Columbia, Florida, Georgia, Maryland, North Carolina, South Carolina, Virginia, West Virginia.

^h^ Alabama, Kentucky, Mississippi, Tennessee.

^i^ Arkansas, Louisiana, Oklahoma, Texas.

^j^ Arizona, Colorado, Idaho, Montana, Nevada, New Mexico, Utah, Wyoming.

^k^ Alaska, California, Hawaii, Oregon, Washington.

^l^ California, Connecticut, Indiana, Louisiana, Maine, Maryland, Minnesota, Montana, New Hampshire, New Mexico, New Jersey, New York, Oklahoma, Oregon, Pennsylvania, Rhode Island, Vermont, Virginia, Washington.

^m^ Alaska, Arizona, Arkansas, Colorado, Delaware, Hawaii, Idaho, Illinois, Iowa, Kentucky, Massachusetts, Michigan, Nebraska, Nevada, North Dakota, Ohio, Utah, West Virginia.

^n^ Alabama, Florida, Georgia, Mississippi, North Carolina, South Carolina, Texas, Wisconsin, Wyoming.

^o^ Kansas, Missouri, South Dakota, Tennessee.

## Discussion

In this nationally representative, serial cross-sectional study, we found evidence of an increase in the number of barriers to reproductive health services among US women of reproductive age between August 2017 and December 2021. Although our survey did not identify the reason for this increase, there were several notable changes in the landscape toward reproductive health care, and health care in general, during this period, including the onset of the COVID-19 pandemic in spring 2020 and significant reductions in the number of Title X family planning clinics in 2019.^21^

One of the greatest increases was observed for barriers pertaining to access (finding a clinic that offered services, finding a clinic where people spoke the same language as the participant, and finding a clinic where the participant felt comfortable). This finding is consistent with results from previous studies,^5,6,7^ suggesting that difficulty finding reproductive health clinics may be a persistent and pressing barrier. An increase in difficulty finding a clinic where participants felt comfortable also aligns with previous research highlighting patients’ fears and anxieties associated with going to a physician or clinic due to uncertainty and fears of contracting COVID-19.^14,22,23,24^ Classifications of reproductive health services as nonessential during the height of the lockdowns^2,25^ also may have contributed to an increased inability to find a clinic offering reproductive health services. Furthermore, increasing restrictions on reproductive rights more broadly^26,27^ may have contributed to an increase in access-related barriers. For instance, in 2019, the Trump administration made substantial changes to Title X, which significantly diminished the number of Title X family planning clinics.^28^ Analyses have shown that the number of family planning visits significantly decreased from 2019 to 2020 as a result,^15,28^ which, coupled with other factors, may have played a role in the increasing number of barriers pertaining to finding a clinic that offers reproductive health services.^28^

Additionally, we saw a large increase in logistical barriers, such as finding transportation and/or child care and getting time off work, between 2017 and 2021. An increase in such barriers may be due to the worsening child care crisis in the US, which was further aggravated by the onset of the COVID-19 pandemic.^29,30,31^ The child care workforce has significantly decreased from 2012 to 2020, with a 12% decrease from 2019 to 2020 alone.^29^ This decrease in workforce, coupled with daycare closures and rising costs, has led to families being unable to secure child care,^29^ which may in turn be associated with increased logistical barriers. Separately, studies have found a decline in use of public transportation^32,33^ during the height of the pandemic, which may have contributed to difficulties in finding transportation to a physician or clinic.

Telemedicine has increasingly been offered as an alternative means of access to overcome such logistical constraints, with Steenland et al^34^ finding significant rates of telemedicine use for contraceptive visits among Medicaid and non-Medicaid patients. Still, some evidence highlights inequities in who was able to use telemedicine. Findings from a nationally representative survey showed that individuals without insurance and those living in regions with limited broadband coverage were less likely to use telemedicine.^35^ However, many reproductive health services cannot be accomplished via telemedicine, such as Papanicolaou tests and insertion and removal of long-acting reversible contraceptive methods.

Interestingly, we found a decrease in the number of people who reported difficulty paying for reproductive health services. Although COVID-19 created many financial hardships, analyses have shown that the issuing of stimulus checks (3 one-time payments ranging between $600 and $1400 each in 2020 and 2021) may have eased some financial burdens and freed up funds for health care spending.^36,37^

We also found that participants identifying with historically marginalized groups frequently experienced the greatest increase in the mean number of barriers to reproductive health services. For example, participants with no high school diploma or General Educational Development test, those living at less than 100% of the FPL, and those born outside of the US who took the surveys in Spanish experienced the largest increase in mean number of barriers from 2017 to 2021. Our findings suggest that barriers to reproductive health services were pervasive and disproportionately associated with reduced access for individuals identifying with historically marginalized groups in both 2017 and 2021. Our study adds to a growing body of literature highlighting how inequities in access to health care services are seemingly widening.^14,38,39^ Although our study does not make clear the reasons for these inequities, we hypothesize that factors associated with inequities across different reproductive outcomes, such as structural and interpersonal racism,^40,41,42^ may also be at play here. Specific attention needs to be paid to how these factors interact and influence individuals’ and communities’ experiences of barriers to health care services to create effective interventions.

### Limitations

This study has some limitations. Because our study design is cross-sectional, we can only describe changes between August 2017 and December 2021 and cannot attribute a specific reason to the significant increase in barriers to reproductive health care. Factors such as COVID-19 and decreases in Title X funding and clinic closures were major events between these 2 periods; however, there may be other associated factors, particularly at the regional level. Importantly, the outcome question on barriers to reproductive health services asked participants whether they had faced barriers to care in the past 3 years; thus, there is 1 year of the post period that took place before the COVID-19 pandemic began. Furthermore, although the Ipsos KnowledgePanel is recruited to represent US households, weights are required to account for differential nonresponse to the survey invitation. Comparing our samples with other nationally representative samples suggests that the weights were largely successful (eTable in Supplement 1).

## Conclusions

This nationally representative, serial cross-sectional survey adds to an existing body of evidence^14,39,43,44,45^ that there has been an increase in the number of barriers experienced while trying to access reproductive health services over the past few years, with individuals from historically disadvantaged populations experiencing the largest increases in barriers to care. However, our findings also suggest that significant barriers to reproductive health services existed in 2017. As such, barriers could persist and, perhaps, continue to worsen.

## Notes

Supplement 1. **eTable.** Sociodemographic Profiles of the National Survey of Family Growth, 2017 to 2019, and the Ipsos KnowledgePanel Populations, 2017 and 2021

Supplement 2.
Data Sharing Statement
