# Supplementary material for: Changes in the Frequency and Type of Barriers to Reproductive Health Care Between 2017 and 2021
Source: JAMA Netw Open. 2023 Apr 10;6(4):e237461. doi: 10.1001/jamanetworkopen.2023.7461 (PMC10087056; doi:10.1001/jamanetworkopen.2023.7461)
Supplement: Supplement 1. — eTable. Sociodemographic Profiles of the National Survey of Family Growth, 2017 to 2019, and the Ipsos KnowledgePanel Populations, 2017 and 2021 [file jamanetwopen-e237461-s001.pdf]

Supplemental Online Content

Adler A, Biggs MA, Kaller S, Schroeder R, Ralph L. Changes in the frequency and type of barriers to reproductive health care between 2017 and 2021. *JAMA Netw Open*. 2023;6(4):e237461. doi:10.1001/jamanetworkopen.2023.7461

eTable. Sociodemographic Profiles of the National Survey of Family Growth, 2017 to 2019, and the Ipsos KnowledgePanel Populations, 2017 and 2021

This supplemental material has been provided by the authors to give readers additional information about their work.

© 2023 Adler A et al. *JAMA Network Open.*

eTable. Sociodemographic Profiles of the National Survey of Family Growth, 2017 to 2019, and the Ipsos KnowledgePanel Populations, 2017 and 2021

		National Survey of Family Growth, 2017-19 (N=5557)	Ipsos Sample, 2017 (N=7022)	Ipsos Sample, 2021 (N = 6841)
		Weighted %	Weighted %	Weighted %
Percent of Federal Poverty Level^				
	<100%	21.2	15.2	11.1
	100 to 199%	22.6	16.0	13.6
	≥ 200%	56.2	68.8	72.3
	Missing	--	--	3.3
Age (years)				
	18-19	5.8	4.6	2.8
	20-24	14.8	14.8	14.4
	25-29	17.1	16.8	17.1
	30-34	16.2	16.5	17.6
	35-39	16.0	15.8	16.8
	40-44	14.5	15.2	15.9
	45-49	15.7	16.2	15.3
Race/ethnicity				
	Non-Hispanic white	57.9	57.1	55.1
	Non-Hispanic Black	14.7	13.1	13.9
	Non-Hispanic other	7.0	9.5	10.0
	Hispanic	20.5	20.3	20.9
Language of the survey				
	English	89.9	91.1	93.1
	Spanish	8.0	8.9	6.9
	Other[a]	2.2	--	--
Highest level of education completed				
	< High school (HS)	8.7	10.0	8.2
	HS diploma or GED	25.4	22.8	21.6
	Some college or associate's degree	22.5	32.0	29.3
	College degree	43.5	35.2	40.9
Ever been pregnant				
	Yes	65.8	n/a[&]	57.9
	No	34.2	n/a	41.5
	Refused	--	n/a	0.6

^ NSFG notes that they conduct multiple imputation on those missing household income; we do not perform multiple imputation and present a separate missing category. [&] Not asked in 2017 survey.

© 2023 Adler A et al. *JAMA Network Open.*
