# Supplementary material for: Changes in the Frequency and Type of Barriers to Reproductive Health Care Between 2017 and 2021
Source: JAMA Netw Open. 2023 Apr 10;6(4):e237461. doi: 10.1001/jamanetworkopen.2023.7461 (PMC10087056; doi:10.1001/jamanetworkopen.2023.7461)
Supplement: Supplement 2. — Data Sharing Statement [file jamanetwopen-e237461-s002.pdf]

Data Sharing Statement

Adler. Changes in the Frequency and Type of Barriers to Reproductive Health Care Between 2017 and 2021. *JAMA Netw Open*. Published April 10, 2023.
doi:10.1001/jamanetworkopen.2023.7461

Data
Data available: No

Additional Information
Explanation for why data not available: Given ethical approvals obtained, data will not be posted in a public repository. However, data can be made available upon reasonable request to the corresponding author.
